# Exploring factors related to heart attack complicated with hypertension using a Bayesian network model: a study based on the China Health and Retirement Longitudinal Study

**DOI:** 10.3389/fpubh.2023.1259718

**Published:** 2023-09-13

**Authors:** Haifen Zhang, Xiaotong Zhang, Xiaodong Yao, Qiang Wang

**Affiliations:** ^1^Department of General Practice, Shanxi Provincial People’s Hospital, The Fifth Clinical Medical College of Shanxi Medical University, Taiyuan, China; ^2^Department of Respiratory and Critical Care Medicine, Shanxi Provincial People’s Hospital, The Fifth Clinical Medical College of Shanxi Medical University, Taiyuan, China; ^3^Department of Infectious Disease, Shanxi Provincial People’s Hospital, The Fifth Clinical Medical College of Shanxi Medical University, Taiyuan, China

**Keywords:** Bayesian networks, multimorbidity, related factors, model construction, heart attack, hypertension

## Abstract

**Objectives:**

While Bayesian networks (BNs) represents a good approach to discussing factors related to many diseases, little attention has been poured into heart attack combined with hypertension (HAH) using BNs. This study aimed to explore the complex network relationships between HAH and its related factors, and to achieve the Bayesian reasoning for HAH, thereby, offering a scientific reference for the prevention and treatment of HAH.

**Methods:**

The data was downloaded from the Online Open Database of CHARLS 2018, a population-based longitudinal survey. In this study, we included 16 variables from data on demographic background, health status and functioning, and lifestyle. First, Elastic Net was first used to make a feature selection for highly-related variables for HAH, which were then included into BN model construction. The structural learning of BNs was achieved using Tabu algorithm and the parameter learning was conducted using maximum likelihood estimation.

**Results:**

Among 19,752 individuals (9,313 men and 10,439 women) aged 64.73 ± 10.32 years, Among 19,752 individuals (9,313 men and 10,439 women), there are 8,370 ones without HAH (42.4%) and 11,382 ones with HAH (57.6%). What’s more, after feature selection using Elastic Net, Physical activity, Residence, Internet access, Asset, Marital status, Sleep duration, Social activity, Educational levels, Alcohol consumption, Nap, BADL, IADL, Self report on health, and age were included into BN model establishment. BNs were constructed with 15 nodes and 25 directed edges. The results showed that age, sleep duration, physical activity and self-report on health are directly associated with HAH. Besides, educational levels and IADL could indirectly connect to HAH through physical activity; IADL and BADL could indirectly connect to HAH through Self report on health.

**Conclusion:**

BNs could graphically reveal the complex network relationship between HAH and its related factors. Besides, BNs allows for risk reasoning for HAH through Bayesian reasoning, which is more consistent with clinical practice and thus holds some application prospects.

## Introduction

Multimorbidity, a condition characterized by the presence of two or more chronic conditions in an individual, has emerged as a major challenge for healthcare services globally (1). This is primarily because multimorbidity is associated with increased healthcare expenditures, decreased quality of life, and reduced life expectancy. In the context of China, the prevalence of multimorbidity reached 42% among the surveyed participants, underscoring its substantial prevalence (2). Moreover, the prevalence and patterns of multimorbidity display noteworthy variances influenced by gender and residential regions within the older Chinese population. The prevalence escalates from 30.2% among individuals aged 50–54 years to 57.5% among those aged 80–84 years (2). Furthermore, a comparative analysis reveals that individuals afflicted with cardiometabolic or respiratory multimorbidity experience markedly escalated risks of mortality, with a similar trend observed for those with gastrointestinal and hepatorenal multimorbidity. Notably, each additional disease is associated with a 36% increase in mortality risk (3).

Despite its growing severity, the prevention of multimorbidity remains a poorly understood area. However, recent research has suggested that psychosocial and behavioral factors, along with population-level interventions and structural changes, may offer effective solutions (4). Addressing risk factors such as smoking, poor diet, and physical inactivity can help to alleviate the burden of multimorbidity.

In the previous work (5), scholars have explored various factors related to multimorbidity, but few have focused on the most common disease combinations. Among these, the combination of heart attack and hypertension (HAH) represents a significant burden on healthcare systems, leading to reduced quality of life and shortened life expectancy. The multifaceted etiology of heart attack complicated with hypertension (HAH) underscores the importance of exploring related factors comprehensively. Previous studies have shown that hypertension is an important risk factor for cardiovascular disease (6), not only accelerating the progression of heart disease but also increasing the susceptibility to develop HAH. Additionally, aging further amplifies susceptibility to cardiovascular disease and requires exhaustive exploration of its complex interactions with other risk factors (7). Besides, lifestyle factors, particularly physical activity and sleep duration, play a key role in maintaining cardiovascular health (8). These factors, together with socioeconomic determinants such as education (9) and functional ability (10), exert a noteworthy influence on the risk of HAH. Understanding the multifaceted relationships between these factors is essential for effective risk assessment and intervention strategies.

Recently, scholars (11–13) employed logistic regression to detect risk factors for hypertension or heart disease. However, these models have limitations, including the inability to account for correlations between variables, make sequential predictions or identify direct and indirect risk factors. Bayesian networks (BNs), proposed by Pearl Judea in 1986, provide a better solution for exploring complex relationships between diseases and their risk factors. BNs consist of a directed acyclic graph (DAG) that reflects potential relationships among influencing factors and a conditional probability distribution table (CPT) that demonstrates correlations between variables.

Compared to traditional models, BNs offer several advantages, including the ability to infer the probability of unknown nodes and flexibly demonstrate the impact of relevant risk factors on HAH. However, to the best of our knowledge, no study has yet employed BNs for exploring risk factors for HAH. This is a significant gap in the literature, as HAH is a major cause of morbidity and mortality worldwide.

Tabu-Search, a global optimization algorithm introduced by Glover (14), allows for good structure learning for BNs, overcoming the limitations of traditional logistic regression. Now, BNs has been applied to explore risk factors for stroke (15), hyperlipidemia (16), hyperhomocysteinemia (17). To the best of our knowledge, no scholars have yet sought to employ BNs for related factor exploration in HAH. If applied, it would be clinically significant.

To this end, this study aims to employ BNs with the Tabu algorithm to detect risk factors for HAH using data from the China Health and Retirement Longitudinal Studies (CHARLS) 2018 involving 19,752 participants. We believe the findings of this study will provide new insights for clinical practice and reduce the prevalence of HAH. By identifying the most important risk factors for HAH, healthcare providers can develop targeted interventions to prevent and manage this condition, improving patient outcomes and reducing healthcare costs.

## Materials and methods

### Study participants

The China Health and Retirement Longitudinal Studies (CHARLS) is an ongoing longitudinal survey that aims to provide a comprehensive understanding of the social, economic, and health conditions of middle-aged and older individuals aged 45 or above in China. The baseline survey was initiated in 2011 and is followed up every 2 years. Covering 150 districts and 450 urban and rural communities across China, with approximately 10,000 households and 17,000 participants, the study provides valuable insights into the collective situation of middle-aged and older people in China (18).

For this study, data was obtained from the fourth published dataset of CHARLS, which was made available on September 23, 2018.^1^ The study’s inclusion criteria encompassed individuals aged 45 years and above and the utilization of data sourced from the CHARLS dataset. Exclusion criteria were applied stringently, targeting severely incomplete data pertaining to essential variables. Furthermore, the exclusion criteria extended to participants whose data lacked crucial demographic information such as age, gender, and household registration. All respondents provided informed consent, and the Institutional Review Committee of Peking University approved all CHARLS waves. The survey was conducted in nine randomly selected provinces using a multi-stage stratified group random sampling method in the east, center, and west of China. The questionnaire survey gathered information related to Family Transfer, Family Information, Work Retirement, Pension, and Household Income of the population who participated in the survey in 2018. The selection of independent variables in this study was guided by a rigorous and well-founded approach. These variables were chosen based on their known associations with cardiovascular health and multimorbidity, as well as their potential to contribute to the intricate network underlying HAH. Some variables had missing values, which were handled using multiple imputation with the RF method with package “mice” in R software.

### Variable definition

#### General information

Age is categorized as <55 years, 55–65 years, 65–75 years, and > 75 years. Marital status is classified as Married, Divorced, Widowed, or Never Married. Educational background is categorized as incomplete primary school (≤ primary school), primary school/junior high school (≤ high school), high school/secondary school/junior college (< college), undergraduate and above (≥ college). Residence is classified as Town, Combination zone between urban and rural areas (boundary), Village, or Special area. Smoking and alcohol consumption are classified as “No” and “Yes.” Sleep duration is categorized as ≤5 h, 5–6 h, 6–7 h, 7–8 h, and ≥ 8 h. Asset is defined as ≤3,000 Yuan, ≤8,000 Yuan, or >8,000 Yuan. Self-reported health comprises very poor, poor, fair, good, and very good. Internet access is defined as “No” or “Yes.”

#### Activity of daily living assessment

The assessment consists of Basic Activities of Daily Living (BADL) and Instrumental activities of daily living (IADL). BADL includes six parameters: dressing, bathing, eating, going to bed, going to the toilet, and controlling defecation. IADL comprises six parameters: doing housework, preparing meals, shopping, managing money, and taking medicine. Any indicator that is unable to be completed is defined as impaired BADL or IADL (19).

#### Physical activity

The International Physical Activity Questionnaire (IPAQ) (20) was used to assess physical activity. Energy expenditure was calculated using the exercise intensity assigned to the physical activity, weekly frequency (days/week), and time per day (minutes/day). The sum of energy expenditure of the three intensity types was the total physical activity expenditure of a week. Vigorous intensity physical activity was assigned a value of 8.0, moderate intensity 4.0, and walking 3.3. Physical activity was categorized into three mutually exclusive groups based on the calculated physical activity energy expenditure: low (<600 MET-min/week), moderate (600–3,000 MET-min/week), and high (≥3,000 MET-min/week).

The CHARLS database collected information on doctor-diagnosed chronic diseases, including 14 types of diseases, by asking “Have you been diagnosed by a doctor with any of the following diseases”: hypertension, dyslipidemia, diabetes, cancer, chronic lung disease, liver disease, heart disease, stroke, kidney disease, stomach or digestive system disease, emotional or psychiatric problems, memory-related diseases, arthritis, or rheumatism, and asthma. In this study, heart attack in combination with hypertension (HAH) was defined as the response variable.

### Elastic Net

Lasso regression is not appropriate for variables with multi-linearity as it does not take into account the correlations between features. Similarly, Ridge regression cannot create a model with a coefficient of zero, which makes it unsuitable for feature selection. In 2005, Zoo and Hastie (21) proposed the Elastic Net (EN) penalty model, which is a combination of Lasso and Ridge regression. The EN regression estimates can be expressed as a convex combination of Lasso and Ridge regression, given by the following equation:


$$\hat{B}=\arg\begin{array}{c} \min\\ \beta^{2} \end{array}∥Y-X\beta {∥}_{2}^{2}+\lambda \left(\alpha ∥B{∥}_{1}+\frac{\left(⊢\alpha \right)}{2}∥B{∥}_{2}\right)\;\left(1\right)$$

Here, β is the regression coefficient, and λ represents the penalty coefficient. α is a value that ranges from 0 to 1, and it adjusts the penalty with λ . When α = 1, the EN model is equivalent to Lasso regression, and when α = 0, it is equivalent to Ridge regression. By combining the two, EN regression can create a sparse model with ideal feature selection, compensating for the effects of correlation between observed variables.

### Bayesian networks

BNs (22) is a Directed Acyclic Graph (DAG) proposed by Judea Pearl in1988. It consists of nodes representing variables and directed edges connecting them. Nodes represent random variables and direct edges between nodes represent the interrelationship between nodes (from the parent node to its child nodes). It uses a conditional probability distribution table (CPT) to express the strength of the relationship quantitatively. When this is no parental node, it was expressed with prior probabilities.

Thus, BNs use the graphical structure and network parameters to uniquely determine the joint probability distribution on the random variable which can be listed as:


$$\begin{array}{c} P\left(x_{1},x_{2},\cdots ,x_{n}\right)=P\left(x_{1}\right)P_{\left(x_{2}|x_{1}\right)}\cdots P\left(x_{n|x_{1},x_{2,}\cdots ,x_{n-1}}\right)\left(2\right) \end{array}$$

$$=\Pi_{1}^{n}P\left(x_{i}|\pi \left(x_{i}\right)\right)$$

In the Bayesian network, let $\pi \left(x_{i}\right)\;$ represent the set of parent nodes of $x_{i}$ , where $\pi \left(x_{i}\right)\subseteq \left(x_{1},\ldots {\mathrm{,x}}_{i-1}\right)$ . Given the values of $\pi \left(x_{i}\right)$ , $x_{i}$ is conditionally independent of the other variables in $\left(x_{1},\ldots {\mathrm{,x}}_{i-1}\right)$ .

### Statistical analysis

The percentages (%) were used to describe the qualitative data and the Chi-square tests were used to make comparisons. The EN was obtained through the use of the “Glmnet” package, while the structure learning of the BNs was carried out using the tabu function in the “bnlearn” package in R studio (4.2.0). The maximum likelihood estimation was used for parameter learning of the BNs. The Netica software was used to plot the Bayesian reasoning and conditional probability distribution tables. *p* < 0.05 was considered to be statistically significant.

## Results

### Baseline characteristics of respondents

A total of 19,752 participants were included in the study, consisting of 11,382 patients with HAH, with 5,400 males (47.4%) and 5,982 females (52.6%). The proportions of patients in different age groups were 12.7% for those aged <55 years, 30.1% for those aged between 55 and 65 years old, 34.5% for those aged between 65 and 75 years old, and 22.7% for those aged >75 years old. The percentages of participants engaging in light, moderate, and vigorous physical activity were 15.5, 30.8, and 53.7%, respectively. The distribution of education levels was 44.9% for ≤ primary school, 42.8% for ≤ middle school, 11.4% for < college, and 0.9% for ≥ college.

Among the individuals without HAH, 3,913 were male (46.8%) and 4,457 were female (53.2%). The proportions of participants in different age groups were 24.2% for those aged <55 years, 37.1% for those aged between 55 and 65 years old, 27.4% for those aged between 65 and 75 years old, and 11.3% for those >75 years old. The percentages of participants engaging in light, moderate, and vigorous physical activity constituted 10.8, 27 and 62.3%, respectively. The distribution of education levels was 41.5% for ≤ primary school, 45.2% for ≤ middle school, 12.5% for < college, and 0.8% for ≥ college. Detailed statistical description on other variables could be available in Table 1.

**Table 1:** Baseline characteristics of individuals with and without HAH.

Variables	Non-HAH (*N* = 8,370)	HAH (*N* = 11,382)
**Sex**		
Male	3,913 (46.8%)	5,400 (47.4%)
Female	4,457 (53.2%)	5,982 (52.6%)
**Age (years)**		
<55	2025 (24.2%)	1,440 (12.7%)
55 ~ 65	3,105 (37.1%)	3,428 (30.1%)
65 ~ 75	2,293 (27.4%)	3,931 (34.5%)
>75	947 (11.3%)	2,583 (22.7%)
**Physical activity**		
Light	901 (10.8%)	1767 (15.5%)
Moderate	2,256 (27%)	3,508 (30.8%)
Vigorous	5,213 (62.3%)	6,107 (53.7%)
**Educational levels**		
≤ Primary school	3,477 (41.5%)	5,111 (44.9%)
≤ Middle school	3,782 (45.2%)	4,873 (42.8%)
< College	1,045 (12.5%)	1,297 (11.4%)
≥ College	66 (0.8%)	101 (0.9%)
**Residence**		
Town	1,416 (16.9%)	2,180 (19.2%)
Combination	591 (7.1%)	837 (7.4%)
Village	6,333 (75.7%)	8,308 (73%)
Special area	30 (0.4%)	57 (0.5%)
**Marital status**		
Married	7,395 (88.4%)	9,494 (83.4%)
Divorced	111 (1.3%)	130 (1.1%)
Widowed	818 (9.8%)	1,686 (14.8%)
Never married	46 (0.5%)	72 (0.6%)
**Sleep duration (h)**		
<5	2,564 (30.6%)	3,796 (33.4%)
5 ~ 6	1880 (22.5%)	2,435 (21.4%)
6 ~ 7	1,514 (18.1%)	1871 (16.4%)
7 ~ 8	1,679 (20.1%)	2,136 (18.8%)
>8	733 (8.8%)	1,144 (10.1%)
**Nap (h)**		
0	3,407 (40.7%)	4,174 (36.7%)
0 ~ 30	1,456 (17.4%)	2030 (17.8%)
> 30	3,507 (41.9%)	5,178 (45.5%)
**Smoking**		
No	4,972 (59.4%)	6,572 (57.7%)
Yes	3,398 (40.6%)	4,810 (42.3%)
**Alcohol consumption**		
No	5,492 (65.6%)	7,621 (67%)
Yes	2,878 (34.4%)	3,761 (33%)
**Social activity**		
No	3,780 (45.2%)	5,468 (48%)
Yes	4,590 (54.8%)	5,914 (52%)
**Internet access**		
No	7,055 (84.3%)	10,042 (88.2%)
Yes	1,315 (15.7%)	1,340 (11.8%)
**BADL**		
No	7,054 (84.3%)	8,610 (75.6%)
Yes	1,316 (15.7%)	2,772 (24.4%)
**IADL**		
No	6,639 (79.3%)	7,799 (68.5%)
Yes	1731 (20.7%)	3,583 (31.5%)
**Self report**		
Very good	1,269 (15.2%)	1,139 (10%)
Good	1,232 (14.7%)	1,297 (11.4%)
Fair	4,179 (49.9%)	5,419 (47.6%)
Poor	1,325 (15.8%)	2,715 (23.9%)
Very poor	365 (4.4%)	812 (7.1%)
**Asset**		
≤3,000 Yuan	3,267 (39%)	4,938 (43.4%)
≤8,000 Yuan	1,482 (17.7%)	1968 (17.3%)
>8,000 Yuan	3,621 (43.3%)	4,476 (39.3%)

### Elastic Net

**Figure 1:**
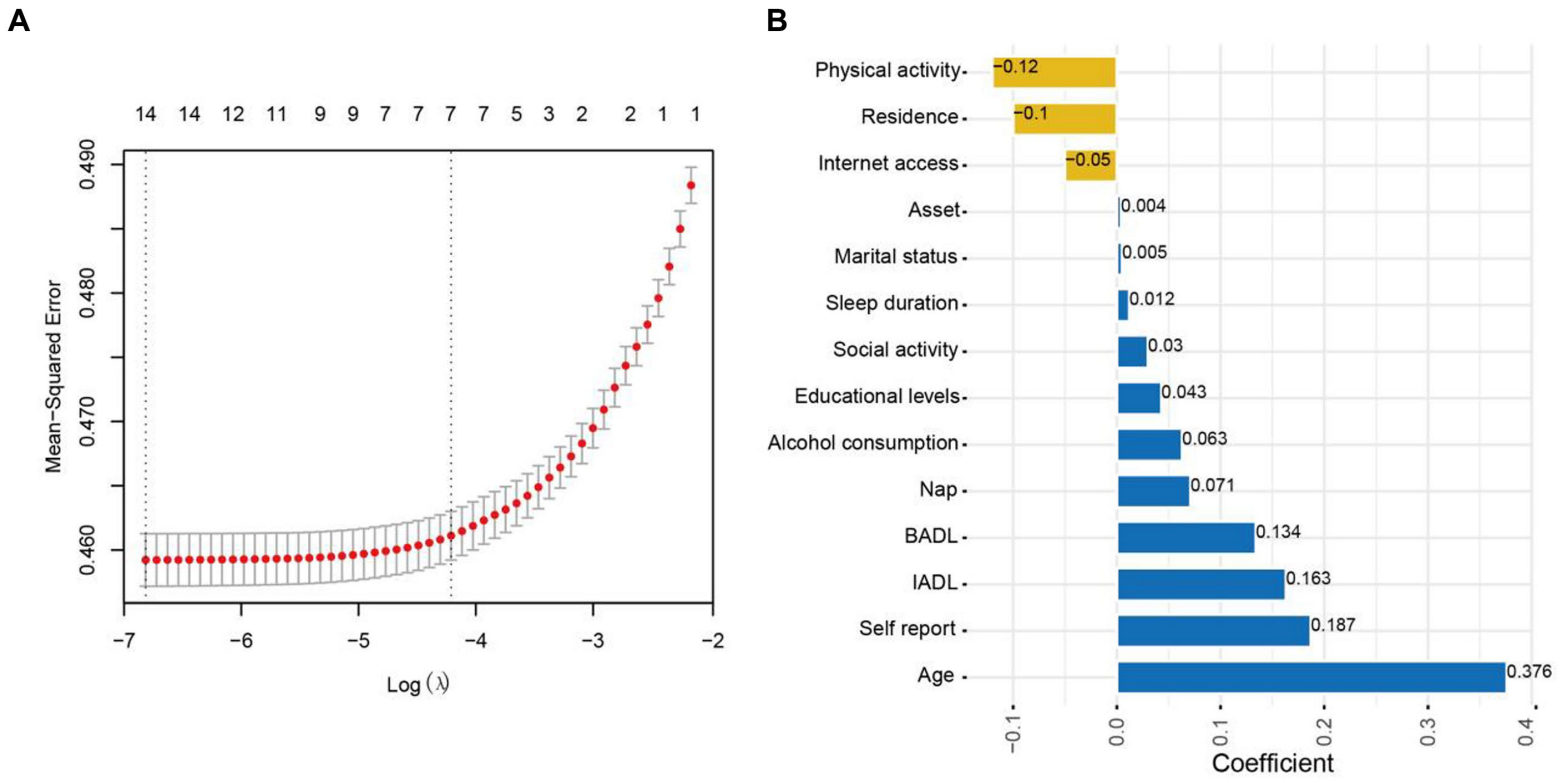
Feature selection using Elastic Net.

In this study, Elastic Net (EN) was used to select the features, and the best model performance was achieved by optimizing the key parameters using tenfold cross-validation (α = 0.9). The coefficients of the risk factors that were not closely related to HAH were compressed to 0 and removed. The analysis found that 14 factors were associated with HAH and included in the BN model establishment. These factors were Physical activity (−0.120), Residence (−0.100), Internet access (−0.050), Asset (0.004), Marital status (0.005), Sleep duration (0.012), Social activity (0.030), Educational levels (0.043), Alcohol consumption (0.063), Nap (0.071), BADL (0.134), IADL (0.163), Self-report on health (0.187), and age (0.376), as shown in Figure 1. This approach simplified the structure of the BNs by focusing on the risk factors that were closely associated with HAH.

### Bayesian networks

**Figure 2:**
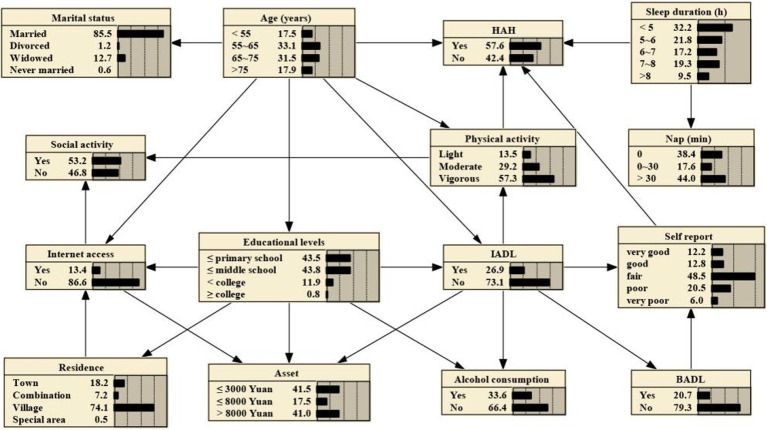
Bayesian networks for HAH and prior probability.

The 14 variables were included in the construction of BNs, which consisted of 15 nodes and 25 directed edges. Each node represents a variable, and the directed edges represent the probabilistic dependence between connected nodes. The number in Figure 2 represents the prior probability of each node, with the prior probability of HAH being 0.576, or P(HAH) = 0.576.

The BNs revealed that age, sleep duration, and physical activity are parental nodes of MMD, indicating a direct association with HAH. Additionally, age is the parental node of both IAD and physical activity, suggesting that age could be directly associated with HAH through IADL and physical activity. Furthermore, age is a parental node of marital status, Internet access, and educational levels, implying that age could be a direct factor related to them. Specifically, educational levels and IADL could indirectly connect to HAH through physical activity, while IADL and BADL could indirectly connect to HAH through self-report on health.

### Bayesian reasonings

BNs can predict the likelihood of disease occurrence by analyzing the influence of various factors on HAH through computing conditional probabilities P(y|xi).

**Figure 3:**
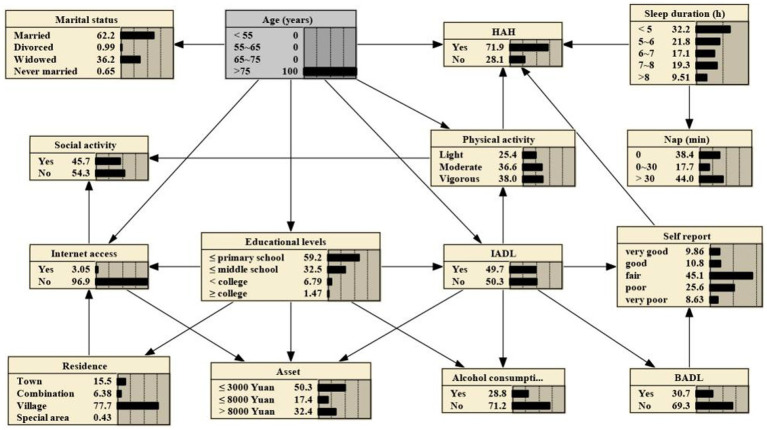
Bayesian networks for HAH for those aged > 75 years.

**Figure 4:**
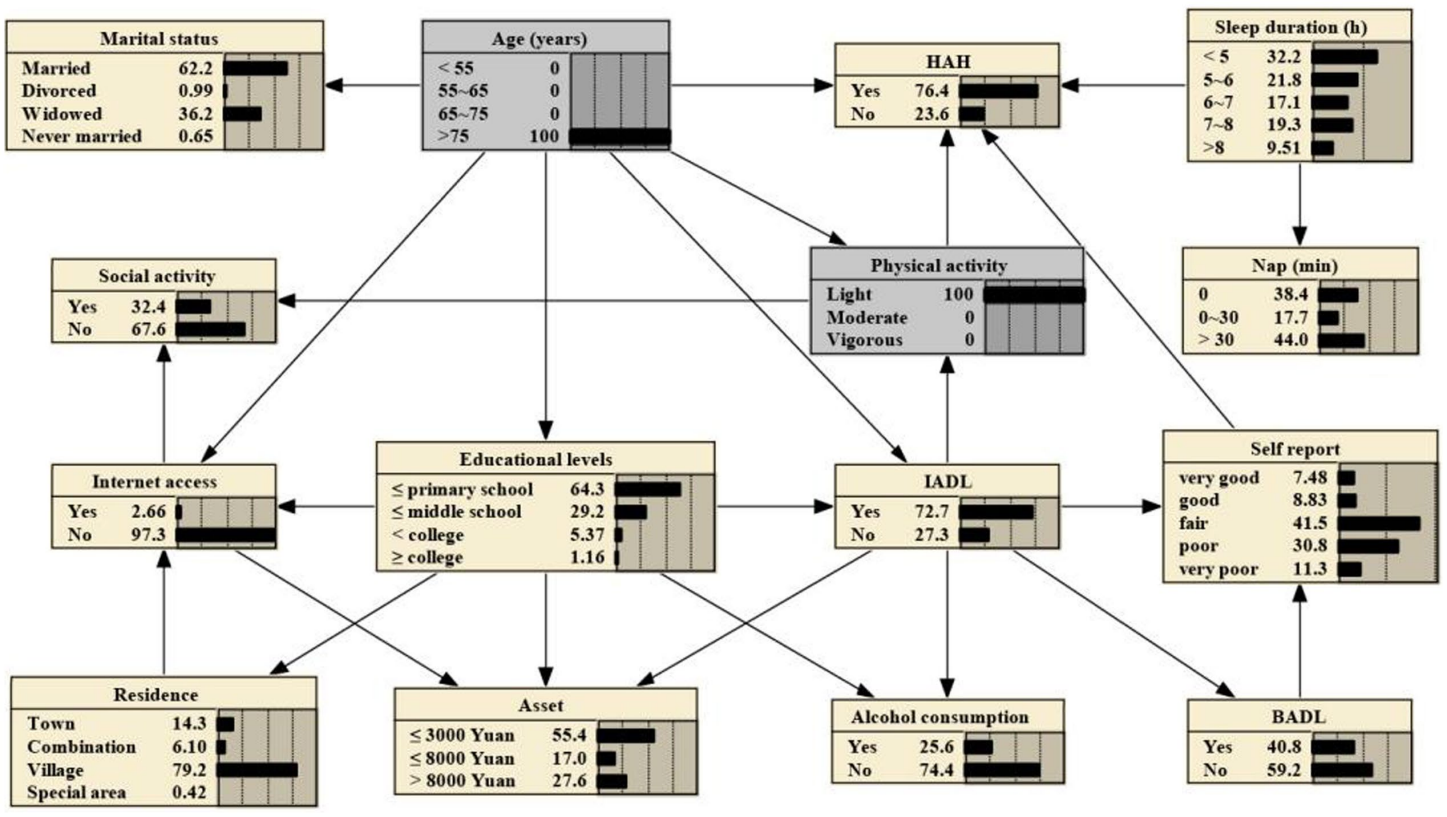
Bayesian networks for HAH for those aged > 75 years with light physical activity.

**Figure 5:**
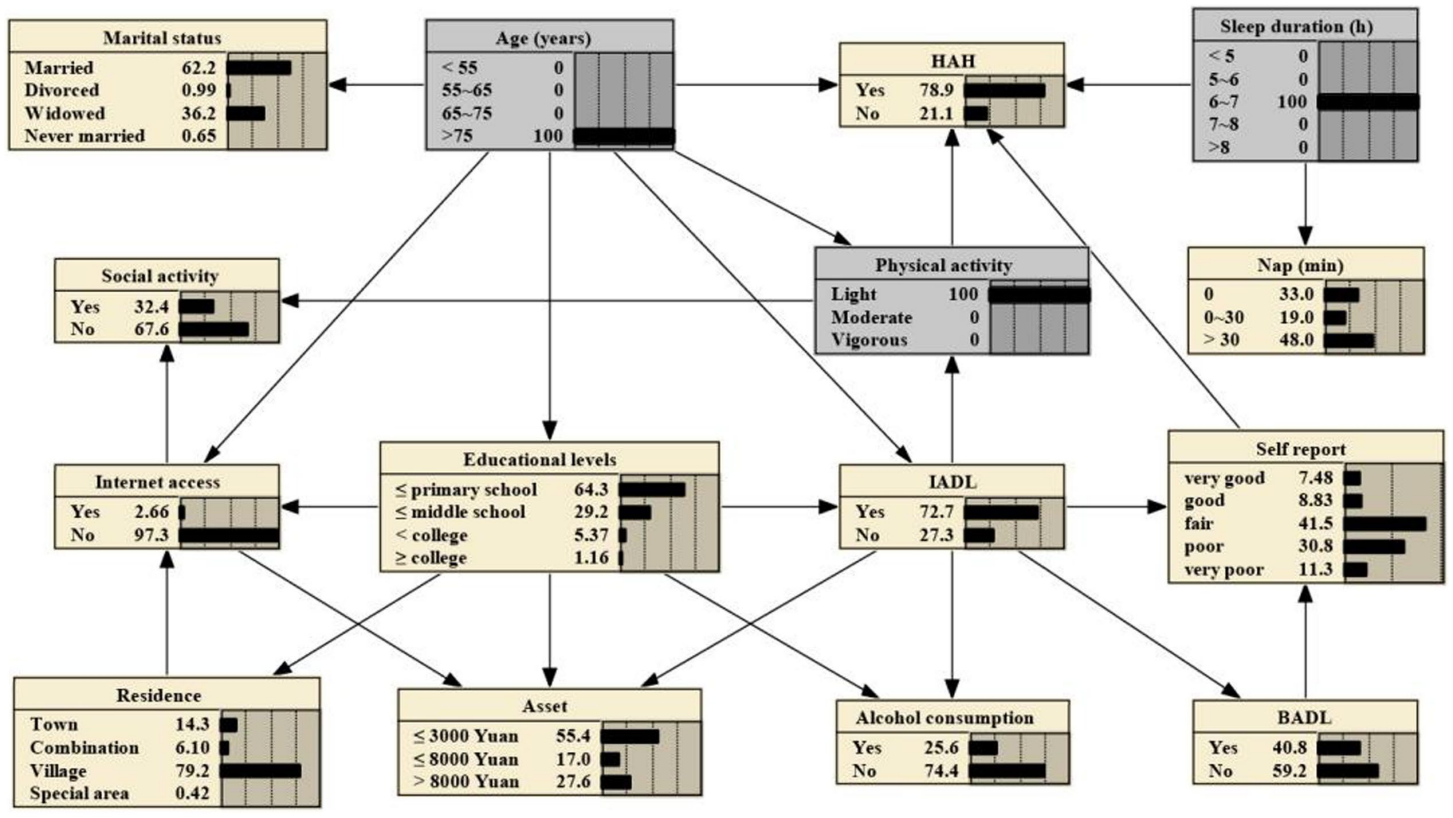
Bayesian networks for HAH for those aged > 75 years with light physical activity and with a sleep duration of 6 to 7 hours.

**Figure 6:**
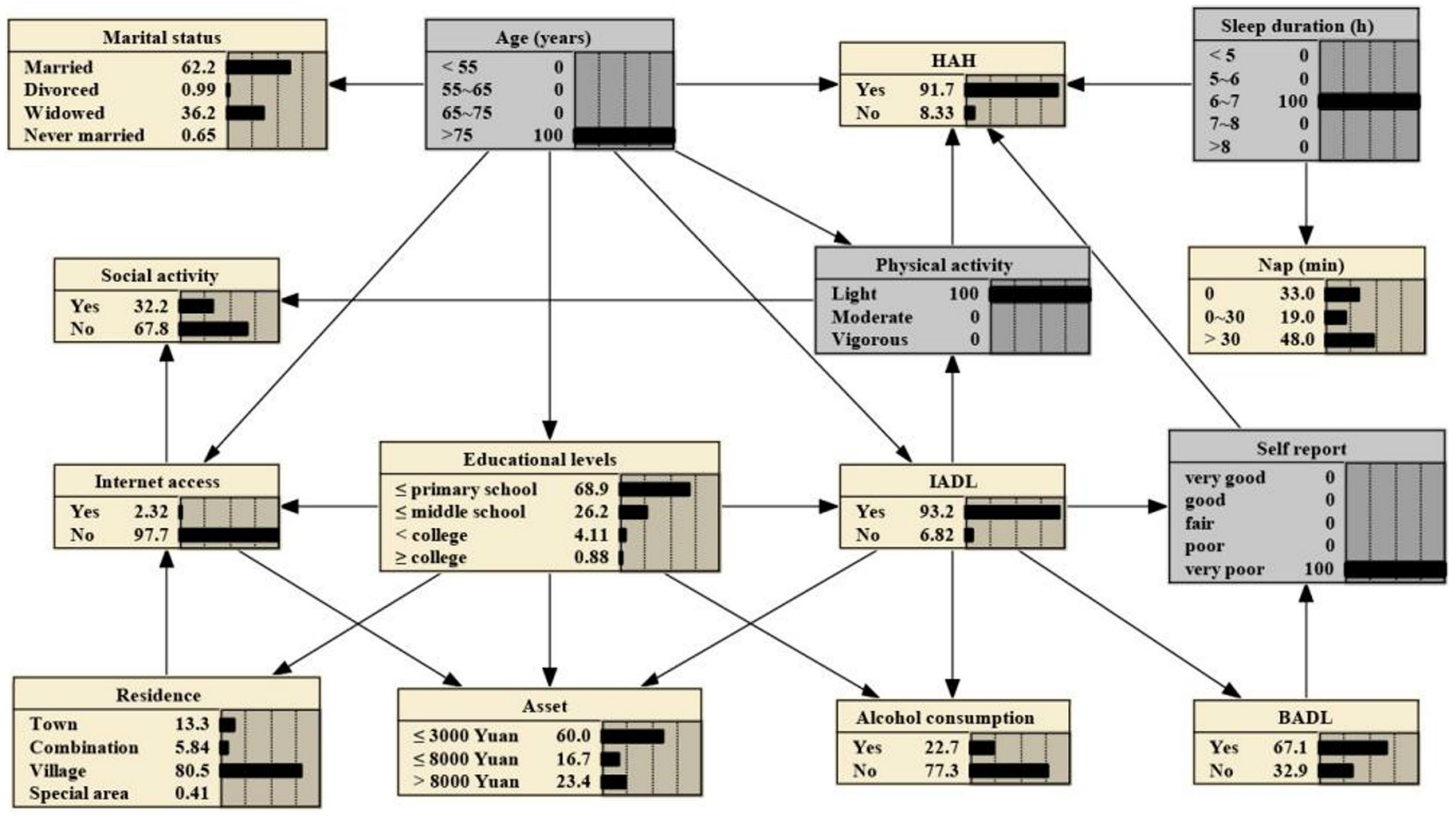
Bayesian networks for HAH for those aged > 75 years with light physical activity, with a sleep duration of 6 to 7 hours and with very poor self report on health.

If one’s age is >75 years, the probability increases from the prior probability to 0.719, that is, P (HAH|age > 75 years) = 0.719, as shown in Figure 3. And if the person is subject to light physical activity, the probability rises to 0.764, that is, P (HAH|age > 75 years, light physical activity) = 0.764, as shown in Figure 4. Besides, If the person’s sleep duration is among 6 to 7 h, the probability rises to 0.789, that is, P (HAH|age > 75 years, light physical activity, sleep duration with 6–7 h) = 0.789, as shown in Figure 5. Additionally, If the person’s self report on health is very good, the probability to develop HAH amounts to 0.917, that is, P (HAH|age > 75 years, light physical activity, sleep duration with 6–7 h, very good self report) = 0. 917, as shown in Figure 6.

## Discussion

When investigating risk factors for HAH, previous studies often relied on logistic regression, which uses probabilities to indicate the strength of the association. However, it cannot provide a comprehensive overview of the overall association between risk factors (15), nor can it detect direct or indirect risk factors. In contrast, BNs offer several advantages over logistic regression in establishing risk factor models (23). Firstly, BNs do not require any prior assumptions. Secondly, the model can integrate multiple variables and analyze their relative importance (24). As a result, BNs have gained popularity among clinical researchers in recent years. Research has demonstrated that BNs, as risk assessment tools for large clinical datasets, can quantitatively identify key indicators for predicting specific histopathological diagnoses and prognoses and identifying risk factors for diseases, which can support medical decision-making (25). It is important to note that the complexity of the network increases as more variables are included in BNs construction; therefore, BNs should be constructed based on both Elastic Net. In this study, we employed BNs with Tabu-search algorithm to explore factors associated with HAH. The findings of this study shed light on the factors associated with heart attack complicated with hypertension (HAH) from a clinical standpoint. The results revealed that several key factors directly influence the occurrence of HAH.

Firstly, age emerged as a key determinant directly related to HAH, confirming the progressive vulnerability of the older adult to cardiovascular disease (26). Older adults are more vulnerable to lifestyle and environmental changes, which may contribute to the onset of hypertension and subsequently increase the risk of developing HAH. In addition, age affects various body systems that directly influence HAH, including vascular function and metabolic regulation (27, 28). This finding highlights not only the need for age-specific monitoring, but also the need for tailored interventions for this high-risk group.

The impact of lifestyle choices, particularly sleep duration and physical activity, on cardiovascular health is widely recognized. Sleep duration was found to have a direct association with HAH (29). Lack of sleep or poor quality sleep disrupts hormonal balance and increases the risk of high blood pressure and heart disease (30). Sleep deprivation triggers overactivity of the sympathetic nervous system, leading to increased blood pressure and cardiovascular stress (31). Besides, sleep deprivation can lead to weight gain, further increasing the risk of cardiovascular disease (32). Optimal sleep duration and quality of sleep play a crucial role in maintaining cardiovascular health and reducing the risk of hypertension. Healthcare providers can use this information to emphasize the significance of healthy sleep habits and address sleep-related issues in patients with HAH. Furthermore, the level of physical activity was identified as another direct factor related to HAH (33). Regular physical activity has well-established cardiovascular benefits. Adequate physical activity helps lower blood pressure, improves blood circulation, and enhances the endurance of the heart muscle, all of which help reduce the risk of hypertension (34, 35). Conversely, lack of physical activity can lead to decreased vascular function and myocardial weakness (36, 37), which can increase the risk of HAH. These findings are consistent with previous studies and highlight the importance of these modifiable factors in shaping cardiovascular outcomes. By weaving these results into existing discourse, we can enhance the applicability of our findings in informing personalized interventions.

Additionally, self-report on health demonstrated a direct association with HAH. Those who perceive themselves to be in poor health may be experiencing underlying physiological problems that can increase the risk of developing hypertensive disorders. Also, psychological distress and poor mental health activate the body’s stress response mechanisms, which may lead to increased blood pressure and cardiovascular strain (38). This relationship emphasizes the importance of considering psychological factors when assessing cardiovascular risk. Addressing a patient’s mental health and well-being can help manage the risk of HAH by reducing stress-induced physiological responses (39). This finding highlights the significance of individuals’ perception of their own health status in relation to HAH.

Moreover, educational levels (9) and instrumental activities of daily living (IADL) were found to have indirect connections to HAH through physical activity. Education and IADL can enable individuals to make informed choices about their lifestyles (10, 40), including participation in activities that contribute to cardiovascular health. In addition, individuals with higher education may have greater access to resources and information related to physical activity. Education level indirectly affects the risk of HAH by influencing the likelihood that an individual will engage in regular physical activity. Clinicians can consider educational levels as an additional factor when assessing an individual’s cardiovascular risk profile.

Lastly, IADL and BADL were found to have indirect connections to HAH through self-report on health. IADL and BADL cover an individual’s ability to perform everyday tasks that reflect their overall physical health. This, in turn, affects how they perceive their health. Impaired functional capacity can lead to reduced quality of life and increased stress, both of which contribute to an increased risk of cardiovascular disease, including hypertension (41, 42). This implies that individuals’ functional abilities and their perception of health status interact to influence the risk of HAH. Healthcare professionals should assess both functional abilities and self-reported health status in patients with HAH to provide comprehensive care and interventions. Overall, these indirect relationships highlight the multifaceted nature of cardiovascular risk factors. They emphasize the importance of considering not only physiological factors, but also mental health, socioeconomic determinants and functional capacity when assessing HAH risk. This holistic assessment allows healthcare professionals to develop tailored interventions that address the underlying mechanisms contributing to HAH risk and ultimately improve patient prognosis.

This study has several limitations that should be taken into consideration when interpreting the findings. Firstly, as a cross-sectional study, it cannot establish a causal relationship between HAH and its risk factors, but only a correlation. Secondly, the data was collected through self-report questionnaires, which may have resulted in an underestimation of the prevalence of HAH, particularly among older people and those with lower socioeconomic and educational backgrounds. Additionally, there may be other variables that were not included in this study. To further enrich our understanding, future studies should consider incorporating environmental variables and exploring the interactions between mental health and HAH. By expanding our study to these dimensions, we can gain a more comprehensive understanding of the multifaceted nature of HAH. Lastly, we did not perform a hierarchical regression model or mediation analysis, which is also an area for future research.

In conclusion, our study demonstrates that BNs, combined with Tabu-search algorithms, is a promising method for identifying risk factors for HAH. This approach could provide valuable insights for clinical practice and medical research. We recommend regular monitoring of chronic diseases to lower the occurrence of HAH and prevent its related diseases.

## Data availability statement

The raw data supporting the conclusions of this article will be made available by the authors, without undue reservation.

## Author contributions

HZ drafted the manuscript and responsible for the conception and design of the research. XZ and XY helped polish the manuscript. QW gave precious advice on the statistical methods. All authors contributed to the article and approved the submitted version.

## Funding

The author(s) declare that no financial support was received for the research, authorship, and/or publication of this article. This paper was supported by the Shanxi Provincial Science and Technology Department Project.

## Conflict of interest

The authors declare that the research was conducted in the absence of any commercial or financial relationships that could be construed as a potential conflict of interest.

## Publisher’s note

All claims expressed in this article are solely those of the authors and do not necessarily represent those of their affiliated organizations, or those of the publisher, the editors and the reviewers. Any product that may be evaluated in this article, or claim that may be made by its manufacturer, is not guaranteed or endorsed by the publisher.

## Footnotes

^1^ http://charls.pku.edu.cn/
